# Health Beyond Health: Insights and Implications from the Fourth National Health Summit of Nepal, 2024

**DOI:** 10.31729/jnma.8875

**Published:** 2025-01-31

**Authors:** Jay Bhushan Jha, Anik Bikram Karki, Sanjeeb Tiwari

**Affiliations:** 1 Nepal Medical Association, Exhibition Road, Kathmandu, Nepal

**Keywords:** *health beyond health*, *health summit*, *Nepal*, *nepal medial association*

## Abstract

The 4^th^ National Health Summit in Nepal, themed "Health Beyond Health," explored the interconnections between health and social, economic, and environmental factors, emphasizing the role of health as a driver of national progress. The summit brought together diverse stakeholders, including policymakers, healthcare professionals, researchers, and civil society leaders, to address transformative health strategies. With subthemes ranging from universal health coverage to the impact of climate change on health, the event highlighted the importance of a holistic, intersectoral approach to health system strengthening. This viewpoint reflects on the summit's outcomes, its inclusive and participatory approach, and the critical need for translating recommendations into actionable strategies to achieve health equity and resilience for Nepal's future.

## INTRODUCTION

"Health Beyond Health" highlights the importance of social, economic, and environmental factors in shaping health outcomes and supporting societal progress through fairness and justice. A holistic approach ensures everyone, regardless of their situation, can live healthy and fulfilling lives.^1^ The Government of Nepal created the Nepal Health Sector Strategic Plan 2023-2030 to guarantee basic health rights and improve citizens' health through fair and sustainable methods.^2^ The World Medical Association (WMA) has worked on issues like pandemics, tobacco use, alcohol abuse, nutrition, obesity, physical inactivity, and drug-resistant tuberculosis.^3^

The 4^th^ National Health Summit (NHS) was organized to discuss and act on these challenges. Nepal Medical Association (NMA) brought together policymakers, healthcare professionals, researchers, media, and government leaders to develop strategies to strengthen the health system and adopt One Health approaches. The summit focused on a holistic, collaborative approach, addressing topics like universal health coverage, health financing, research, climate change, health journalism, crisis resilience, and health education.

This summit marked a significant shift in Nepal's public health efforts, emphasizing health as a key factor in national progress. It went beyond traditional medical approaches to examine how health connects with social, economic, and environmental factors.

## SUB THEMES

There was a formation of Scientific Team with dedicated experts who played a pivotal role in development of summit themes. The process of theme development involved extensive consultations with experts, review of current health challenges and alignment with the national priorities and global health agendas. Initial broad topic of "Health Beyond Health" was the main center around which subthemes were aligned. Finally seven subthemes were extracted from the discussions.

The summit focused on key health topics and their connections to social and environmental issues. **"From Commitment to Action"** reviewed progress since the 3^rd^ NHS, identifying challenges like lack of funding and staff through reports and discussions.^4^ **"Transformative Health Systems and Universal Health Coverage"** explored ways to improve healthcare access for all, using workshops and global guidelines to suggest ideas like digital health and community-based care.^5-7^ **"Research and Collaboration"** highlighted the importance of partnerships between universities, the government, and international groups to create better health policies.^8^ **"Building Resilience for Crises focused"** on lessons learned from COVID-19 and disasters, like improving healthcare systems and supply chains to handle emergencies better.^9^ **"Improving Medical Education"** called for updating training and teaching methods for healthcare workers, using global standards as a guide.^11^ **"Media and Health Awareness"** emphasized the need for better health reporting, training journalists to fight misinformation, and promoting public understanding of health issues.^12^ Lastly, **"Water, Sanitation, Nutrition, and Climate Change"** examined how environmental factors affected health, suggesting joint efforts to improve water quality, reduce infections, and address climate-related health problems.^13,14^

## SESSIONS

**Grand Inauguration and Setting the Tone**

The summit began with an inauguration ceremony attended by key dignitaries, including the Honourable Minister from the Ministry of Health and Population (MoHP), Secretary from the MoHP, Chairperson form Medical Education Commission (MEC), representative form World Health Organization (WHO). Their speeches inspired participants to think beyond traditional health boundaries and emphasized the importance of an intersectoral approach to health. President of World Medical Association (WMA) also congratulated NMA for the conducting the summit. The inaugural session set the stage for the summit's objectives, which aligned closely with the Nepal Health Sector Strategic Plan 2023-2030, aiming to ensure fundamental health rights and improve health outcomes for all citizens through equity-driven and sustainable approaches.

**A Holistic Approach to Health**

The "Health Beyond Health" theme reflected the understanding that health is deeply interconnected with social, economic, and environmental factors. The summit emphasized that achieving health equity requires addressing these determinants holistically. Subthemes covered a wide range of topics, including Universal Health Coverage (UHC), health financing, health research, climate change and its impact on health, health journalism, resilience for health crises, and health education. This approach resonated with global health priorities and underscored Nepal's commitment to integrating innovative and inclusive strategies into its health policies.

**Dynamic and Engaging Sessions**

The daily sessions, commencing at 8:00 AM, featured a diverse mix of keynotes, panel discussions, workshops, and interactive presentations. These sessions provided a platform for participants to share their expertise, discuss challenges, and propose actionable solutions. Topics ranged from the role of digital health in achieving UHC to strategies for building resilience against health crises such as pandemics and natural disasters.

One of the standout sessions focused on health financing, where experts discussed innovative approaches to reduce out-of-pocket healthcare expenditures and improve access to essential health services. Another session on climate resilience in healthcare highlighted the impact of environmental changes on public health and the need for sustainable, multi-sectoral interventions.

**Inclusivity and Representation**

Inclusivity was a cornerstone of the 4^th^ NHS. The summit ensured that voices from grassroots organizations, community health workers, and youth leaders were heard and valued. This inclusivity was evident in sessions that featured community representatives sharing their experiences and challenges, providing invaluable insights into the realities faced by marginalized populations. By incorporating these diverse perspectives, the summit fostered a sense of shared accountability and collective ownership of health policies and initiatives.

**Collaborative and Multi-Sectoral Engagement**

The involvement of international collaborators and representatives from various sectors added depth to the discussions. Policymakers engaged with healthcare professionals to align health policies with ground realities, while researchers presented evidence-based insights to guide decision-making. Media professionals played a crucial role in emphasizing the importance of responsible health journalism in combating misinformation and raising public awareness.

The summit's multi-sectoral approach was particularly evident in sessions addressing climate change and its impact on health. These discussions highlighted the need for collaboration between environmental scientists, public health experts, and policymakers to develop holistic strategies that address waterborne diseases, malnutrition, and the broader implications of climate change on health.

**Outcomes and Way Forward**

The 4th NHS generated significant momentum, setting the stage for transformative changes in Nepal's health sector. Participants left the summit with a renewed sense of purpose and a shared vision for achieving health equity and resilience. Despite its successes, the NHS faces challenges, including limited funding, logistical hurdles, and inconsistent participation from key stakeholders. Addressing these barriers will require stronger political commitment, robust planning, and active engagement with local communities.

To enhance its impact, the NHS could adopt technological innovations such as virtual platforms to broaden participation and inclusivity, especially for stakeholders in remote regions. Additionally, fostering collaborations with global health initiatives can amplify the summit's influence, aligning Nepal's health priorities with international standards.

## CONCLUSIONS

The 4th National Health Summit was not just an event but a movement that reinforced the deep interdependence of health and development. By addressing the social, economic, and environmental determinants of health, the summit positioned Nepal at the forefront of a global shift towards holistic and inclusive health systems. As the country moves forward, the lessons and collaborations from the summit will serve as a guiding light, reminding all stakeholders of the collective action required to build a healthier, more equitable, and sustainable future.
